# Establishment and Characterization of a Topotecan Resistant Non-small Cell Lung Cancer NCI-H460/TPT10 Cell Line

**DOI:** 10.3389/fcell.2020.607275

**Published:** 2020-12-23

**Authors:** Zi-Ning Lei, Qiu-Xu Teng, Wei Zhang, Ying-Fang Fan, Jing-Quan Wang, Chao-Yun Cai, Kimberly W. Lu, Dong-Hua Yang, John N. D. Wurpel, Zhe-Sheng Chen

**Affiliations:** ^1^Department of Pharmaceutical Sciences, College of Pharmacy and Health Sciences, St. John’s University, Queens, NY, United States; ^2^Institute of Plastic Surgery, Weifang Medical University, Weifang, China; ^3^Department of Hepatobiliary Surgery, Zhujiang Hospital of Southern Medical University, Guangzhou, China; ^4^Renaissance School of Medicine, Stony Brook University, Stony Brook, NY, United States

**Keywords:** topotecan, non-small cell lung cancer, NCI-H460/TPT10, multidrug resistance, ABCG2

## Abstract

While topotecan (TPT) is a first- and second-line chemotherapeutic drug in treating lung cancer, the development of drug resistance in tumors still reserves as a major obstacle to chemotherapeutic success. Therefore, a better understanding of the mechanisms of topotecan resistance is critical. In this study, the first topotecan-resistant human non-small cell lung cancer (NSCLC) cell line, termed NCI-H460/TPT10, was established from the parental NCI-H460 cell line. NCI-H460/TPT10 cells exhibited a 394.7-fold resistance to TPT, and cross-resistance to SN-38, mitoxantrone, and doxorubicin, compared to parental NCI-H460 cells. Overexpression of ABCG2 localized on the cell membrane, but not ABCB1 or ABCC1, was found in NCI-H460/TPT10 cells, indicating that ABCG2 was likely to be involved in topotecan-resistance. This was confirmed by the abolishment of drug resistance in NCI-H460/TPT10 cells after *ABCG2* knockout. Moreover, the involvement of functional ABCG2 as a drug efflux pump conferring multidrug resistance (MDR) was indicated by low intracellular accumulation of TPT in NCI-H460/TPT10 cells, and the reversal effects by ABCG2 inhibitor Ko143. The NCI-H460/TPT10 cell line and its parental cell line can be useful for drug screening and developing targeted strategies to overcome ABCG2-mediated MDR in NSCLC.

## Introduction

Lung cancer is the most frequently diagnosed cancer worldwide and is one of the most common causes of cancer-related death (Bray et al., 2018). In the United States, there were 228,150 estimated new cases of lung cancer with 142,670 estimated deaths in 2019 (Siegel et al., 2017). Non-small cell lung cancer (NSCLC) comprises 80–85% of all lung cancers and has a 5-year survival rate of less than 20% (Chen et al., 2014; Osmani et al., 2018). The dominant therapeutic approach to treat patients with advanced NSCLC is chemotherapy (Gridelli et al., 2015). Although treatment is initially effective, conventional chemotherapies often fail due to the acquired and/or intrinsic multidrug resistance (MDR) in tumors, leading to a high risk of local-regional recurrence and distant relapse (Wangari-Talbot and Hopper-Borge, 2013; Iglesias et al., 2018). There have been many attempts to overcome drug resistance to improve chemotherapeutic efficacy and understanding the mechanisms of resistance is a crucial step to solve the issue of MDR. Many mechanisms can be associated with MDR in lung cancers, such as decreased uptake of drugs that enter cells via transporters, increased efflux of drugs from cells, and changes in the cellular response to drugs, including decreased cell apoptosis, altered drug metabolism, mutated drug target, and increased DNA repair (Wangari-Talbot and Hopper-Borge, 2013; Iglesias et al., 2018; Terlizzi et al., 2019). Although MDR can be multifactorial, one of the most widely observed mechanisms has been the increased drug efflux linked to the overexpression of transmembrane ATP-binding cassette (ABC) transporters (Robey et al., 2018).

Human ABC transporters belong to a superfamily comprised of 48 members, of which three are implicated in the majority of MDR observed in lung cancer cells: ABCG2 [breast cancer resistance protein (BCRP)], ABCB1 [P-glycoprotein (P-gp/MDR1)], and ABCC1 [multidrug resistance-associated protein 1 (MRP1)] (Chen et al., 2016; Katayama et al., 2016). These transporters act as drug pump which could flow out a diverse range of chemotherapeutic drugs across cellular membranes, including camptothecin analogs, anthracyclines, tyrosine kinase inhibitors, topoisomerase inhibitors, and antimetabolites (Mao and Unadkat, 2015; Zhang et al., 2015; Fletcher et al., 2016). In particular, ABCG2 overexpression appears to show a significant correlation with decreased efficacy to topoisomerase I inhibitors such as irinotecan, SN-38, and topotecan in NSCLC (Bessho et al., 2006; Nagashima et al., 2006; Ohtsuka et al., 2010). Topotecan (TPT) is a chemotherapeutic agent with a broad spectrum of anticancer efficacy in a variety of cancers like ovarian, cervical, and lung cancers (Hörmann et al., 2012). Topotecan is currently approved by the US FDA for small cell lung cancer (SCLC) clinically first- and second-line therapies (Spigel et al., 2013). It has shown to be efficacious in the treatment of NSCLC with a favorable side effect profile (Vennepureddy et al., 2015). Several clinical trials demonstrated that either oral or IV single-agent topotecan can be used as a second-line therapy for patients with progressed or relapsed NSCLC. Topotecan in IV formulation exhibited moderate antitumor activity at a dose of 1.5 mg/m^2^/day in a Phase II clinical study conducted by Perez-Soler et al. (1996). Oral topotecan at a dose of 2.3 mg/m^2^/day for 5 days every 21 days had also shown promising antitumor activity on advanced stage III or IV NSCLC with mitigation of symptoms in Phase II and Phase III clinical trials (White et al., 2000; Ramlau et al., 2006). Moreover, Phase II clinical trials suggested that topotecan in combination with other anti-cancer drugs, such as paclitaxel and bevacizumab, could be recommended as a first-line treatment for advanced NSCLC patients who could not tolerate standard platinum-based therapy due to side effects (Stathopoulos et al., 2006; Powell et al., 2013). However, drug resistance can be developed in patients with NSCLC treated with topotecan, which may be related to ABCG2 overexpression as topotecan is a substrate transported by ABCG2 (Mo and Zhang, 2012; Zhang et al., 2018).

Drug-resistant cellular models are powerful tools for examining and garnering an in-depth understanding of the MDR phenotype in lung cancers. Therefore, creating new models will be instrumental in designing novel therapeutic approaches to combat MDR in lung cancers. In this study, the first topotecan-resistant human NSCLC cell line, termed NCI-H460/TPT10, was established. Based on the fact that topotecan is a known substrate of ABCG2, it was hypothesized that ABCG2 would be primarily involved in mediating drug resistance in NCI-H460/TPT10. The drug resistance phenotype of NCI-H460/TPT10 and the potential mechanism of drug resistance were characterized.

## Materials and Methods

### Chemicals and Reagents

Topotecan (TPT) was purchased from Chemitek (Indianapolis, IN, United States). The mouse monoclonal anti-P-glycoprotein antibody, and 3-(4, 5-dimethylthiazol-yl)-2, 5-diphenyltetrazolium bromide (MTT), and dimethyl sulfoxide (DMSO), were supplied from MilliporeSigma Co. (St. Louis, MO, United States). Dulbecco’s modified Eagle’s medium (DMEM), 0.25% trypsin, and fetal bovine serum (FBS) were purchased from Corning Inc. (New York, NY, United States). The rabbit monoclonal antibodies against human MRP1/ABCC1 and human DNA topoisomerase I, the HRP-labeled goat anti-rabbit secondary antibody, and the HRP-linked rabbit anti-mouse secondary antibody were supplied from Cell Signaling (Danvers, MA, United States). Ko143, mitoxantrone (MX), cisplatin, and geneticin (G418) were obtained from Enzo life Sciences (Farmingdale, NY, United States). The mouse monoclonal antibodies for ABCG2 and glyceraldehyde phosphate dehydrogenase (GAPDH), the Alexa Fluor 488-labeled rabbit anti-mouse secondary antibody, 4,6-diamidino-2-phenylindole (DAPI), and all other chemicals were obtained from Thermo Fisher Scientific Inc. (Rockford, IL, United States).

### Cell Lines and Cell Culture

Drs. Susan E. Bates (Columbia University, New York, NY, United States) and Robert Robey (NIH, Bethesda, MD, United States) kindly provided the NSCLC cell line NCI-H460. The HEK293 cells were used to develop series HEK/ABCG2, HEK/ABCB1, and HEK/ABCC1 transfected overexpressing cell lines, which were transfected with the vector containing full-length ABCG2, ABCB1, and ABCC1 DNA, respectively. The empty pcDNA3.1 vector transfected HEK293 cells, termed as HEK293/pcDNA3.1, was used as vector control cell line. They were cultured in medium supplemented with 2 mg/mL of G418 and then were used for Western blotting. All cell lines were cultured in DMEM with 10% FBS in a humidified incubator supplied with 5% CO_2_ at 37°C.

### Establishment of a Topotecan (TPT) Resistant NCI-H460 Cell Line

The topotecan resistant cell line, NCI-H460/TPT10, was generated by continuously maintaining NCI-H460 cells in complete culture medium containing topotecan in gradually increasing concentrations. To be detailed, the parental NCI-H460 cells were first cultured in DMEM with 0.1 μM topotecan at 37°C for 72 h, followed by replacing with fresh drug-free medium. The remaining cells were passaged and cultured in medium containing 0.1 μM topotecan until they stabilized. The concentration of topotecan was increased stepwise up to 10 μM, with a total of 5 months of culturing. The established NCI-H460/TPT10 cells were cultured in the topotecan-free medium for 12 weeks prior to further experiment. The newly established NCI-H460/TPT10 and the parental NCI-H460 cell lines were subjected to Short Tandem Repeat (STR) profile analysis performed by ATCC cell line authentication service. The identity of NCI-H460 cell line and the origin of NCI-H460/TPT10 cell line from NCI-H460 were confirmed from the STR profiles (Supplementary Table 1).

### Population Doubling Time (PDT) Assessment

Cells were seeded to 24-well plates at 5000 cells/well and cultured at 37°C with 5% CO_2_. Triplicate wells were used for each determination. The cells were collected and counted every 24 h for 7 days. Trypan blue exclusion method was used to determine the number of live cells and cell stained with trypan blue were excluded from the counting. The cell growth curve was plotted as log(N) versus time, where N is the average cell count. The linear portion of the cell growth curve, which represents exponential growth, was subjected to PDT calculation using the following equation: PDT = T × log (2)/log (N1/N0). In this equation, T is the culture time, N1 is the cell number at the end of the culture period, N0 is the cell number at the beginning of the culture period.

### Drug Resistance Profile of NCI-H460/TPT10

The MTT colorimetric assay was performed to measure the sensitivity of the resistant cell line NCI-H460/TPT10 to anticancer drugs. Briefly, 4000 cells/well were evenly seeded into 96-well plates and cultured overnight. Cells in assigned wells were added with different concentrations of anticancer drugs and incubated for 68-h, followed by addition of 4 mg/mL MTT and a further 4-h incubation. The medium in each well was subsequently replaced by 100 μL of DMSO. The absorbance of each well at 570 nm was determined by the accuSkan GO UV/Vis Microplate Spectrophotometer to measure the concentration of formazan.

### Western Blotting Analysis

The cell lysates extraction, the protein concentration determination, and the gel electrophoresis were performed as described previously (Zhang et al., 2017). The dilutions of both primary and secondary antibodies are 1:1000 with the blocking agent.

### Immunofluorescence Assay

NCI-H460 and NCI-H460/TPT10 cells were seeded at a density of 100000 cells/well in 24-well plates and incubated overnight. Then, cells were washed twice with ice-cold PBS, followed by fixation using 4% paraformaldehyde, permeabilization using 0.25% Triton X-100 and immunofluorescence assay procedures as previously described (Wu et al., 2020). Immunofluorescence images were taken with an EVOS FL Auto Imaging System (Thermo Fisher Scientific Inc., Rockford, IL, United States).

### Reversal Effects Against Drug Resistance

In order to verify the hypothesis that ABCG2 is involved in mediating drug resistance in NCI-H460/TPT10, a known ABCG2 inhibitor, Ko143 (Mao and Unadkat, 2015), was used to evaluate whether the cells could be re-sensitized to the substrates of ABCG2 in NCI-H460/TPT10. NCI-H460/TPT10 cells and NCI-H460 cells were incubated for 72-h in culture medium containing various concentrations of TPT, with the addition of 3 μM Ko143 2 h ahead or without Ko143. The MTT colorimetric assay, as described in section “Drug Resistance Profile of NCI-H460/TPT10,” was used to assess the reversal effect of ABCG2-mediated topotecan resistance by Ko143 in NCI-H460/TPT10 cell line.

### Topotecan Intracellular Accumulation Assay

The intracellular accumulation of topotecan in NCI-H460/TPT10 and the parental NCI-H460 cells was determined by flow cytometric analysis. Cells were harvested by trypsinization and washed with PBS. Then cells (1 × 106/mL) were incubated at 37°C in culture medium with or without 3 μM Ko143 for 2-h prior to an additional 2-h incubation with culture medium containing 100 μM topotecan with or without 3 μM Ko143. When the incubations were completed, cells were washed twice and resuspended using ice-cold 0.5% bovine serum albumin (BSA) prepared in PBS. Samples were kept on ice to avoid further ABCG2-mediated efflux until flow cytometry analysis was performed. Each sample was subjected to flow cytometry on BD Accuri C6 (BD Biosciences, San Jose, CA, United States). Intracellular topotecan was estimated by relative fluorescence intensity, which was calculated by (mean FL2-H unit of cells in 100 μM topotecan with or without Ko143 – mean FL2-H unit of untreated cells with or without Ko143)/(mean FL2-H unit of parental cells in 100 μM topotecan – mean FL2-H unit of untreated parental cells).

### Construction of NCI-H460/TPT10 ABCG2 Knockout Cell Line

The ABCG2 gene knockout subline of NCI-H460/TPT10 was constructed using a clustered regularly interspaced short palindromic repeats (CRISPR)/CRISPR-associated (Cas) 9 system. The custom-designed mammalian CRISPR vector and its control vector were purchased from VectorBuilder Inc. (Chicago, IL, United States). The vector consists of a U6 promoter that regulates the transcription of guide RNA (gRNA), a CBh promoter that regulates the expression of Cas9 nuclease, and a CMV promoter that regulates the transcription of the neo gene responsible for resistance to G418. The gRNA of the human ABCG2 targeting vector contains a specific 20 bp guide sequence of 5′-GATCATTGTCACAGTCGTAC-3′ selected from exon 10 of human ABCG2 gene and a scaffold sequence specific for Cas9 protein. The same vector with target sequence in gRNA replaced by a scramble sequence of 5′-GTGTAGTTCGACCATTCGTG-3′ was used as the negative control. In this study, NCI-H460/TPT10 cells were transfected with either the targeting vector or the control vector using Fugene6 transfection reagent (Madison, WI, United States) following the manufacturers’ protocol. With changing the medium every third day, the transfected cells were incubated with the selection medium for 14 days. Then, single positive colonies were obtained by limited dilution. Measurement of protein expression using western blotting was conducted to verify the knockout of ABCG2 (Supplementary Figure 1). The NCI-H460/TPT10-ABCG2 knockout subline and the vector control were further used in drug sensitivity tests to topotecan, mitoxantrone, SN-38 and cisplatin by MTT assay.

### Statistical Analysis

Student’s *t*-test was used in comparing parental and resistant cell lines in drug-resistant profile. All other statistical analysis comparing among multiple groups was performed by one-way ANOVA followed by Tukey *post hoc* test. All the statistical analysis was carried out in GraphPad Prism 8 (GraphPad Software, La Jolla, CA, United States). Statistical significance was set at *p* < 0.05. All data subjected to statistical evaluations were gathered from at least three independent repeats of experiments.

## Results

### Establishment of the Topotecan-Resistant Cancer Cell Line and Drug-Resistant Profile

The topotecan-resistant NSCLC cell line NCI-H460/TPT10 was eventually developed by selecting the parental NCI-H460 cells in stepwise increasing concentrations of topotecan until cells survive in topotecan at the concentration up to 10 μM. To compare the exponentially growing cells rate of NCI-H460/TPT10 and its parental cell line. The PDT (the amount of time that the cells takes to double their population) was calculated. The PDT of NCI-H460/TPT10 subline was 22.46 ± 0.85 h and NCI-H460 cell line had a PDT of 18.39 ± 1.35 h. The growth curves were shown in Supplementary Figure 2. Although the PDT of the resistant cell line was slightly longer than the parental cell line, the difference is not statistically significant (*p* = 0.07 by Student’s *t*-test).

The drug resistant profile of NCI-H460/TPT10 was summarized in Table 1. The topotecan IC_50_ values determined for NCI-H460 cells and NCI-H460/TPT10 cells were 83.076 ± 9.989 nM and 32789.712 ± 3618.593 nM, respectively. NCI-H460/TPT10 cells exhibited a 394.7-fold resistance to topotecan compared to NCI-H460, indicating a resistance-mediated improvement in survival. Besides, NCI-H460/TPT10 conferred 176.9- and 172.6-fold cross-resistant to typical ABCG2 substrates SN-38 and mitoxantrone, respectively, which suggested that the human ABCG2 transporter might be a factor mediating the drug resistance in this new cell subline. Moderate cross-resistance (8.5-fold) to doxorubicin, which is a substrate of both ABCG2 and ABCB1, was exhibited in NCI-H460/TPT10 cells. However, the IC_50_ values of other ABCB1 or ABCC1 substrates, including paclitaxel, colchicine, and vincristine, were not significantly different between NCI-H460/TPT10 and its parental cells. Also, NCI-H460/TPT10 did not show resistance to cisplatin, which is not a substrate of ABCB1, ABCC1, and ABCG2 transporters.

**TABLE 1:** The drug-resistant profile of NCI-H460/TPT10 cell line.

**Anticancer drugs**	**IC_50_ ± SD^a^ (nM)**		**RF^b^**
	**NCI-H460**	**NCI-H460/TPT10**	
Topotecan	83.076 ± 9.989	32789.712 ± 3618.593*	394.7
SN-38	88.561 ± 7.697	15668.540 ± 1326.075*	176.9
Mitoxantrone	33.316 ± 1.636	5748.677 ± 609.914*	172.6
Paclitaxel	8.731 ± 1.575	10.023 ± 2.515	1.1
Doxorubicin	20.194 ± 1.007	172.541 ± 9.478*	8.5
Colchicine	2.519 ± 0.194	2.822 ± 0.036	1.1
Vincristine	28.185 ± 1.619	30.835 ± 2.597	1.1
Cisplatin	811.648 ± 50.606	868.808 ± 85.056	1.1

^*a*^IC_50_: concentration that reduced cell viability by 50%. Values in the table are represented as means ± SD determined from at least three independent experiments performed in triplicate. ^*b*^RF: resistant-fold represents IC_50_ value for different anticancer drugs of NCI-H460/TPT10 cells was divided by IC_50_ value for different anticancer drugs of NCI-H460 cells. *Indicates a significant difference between IC_50_ value of NCI-H460 and that of NCI-H460/TPT10 (*p < 0.05) by Student’s *t*-test.

### Western Blotting Analysis

**FIGURE 1:**
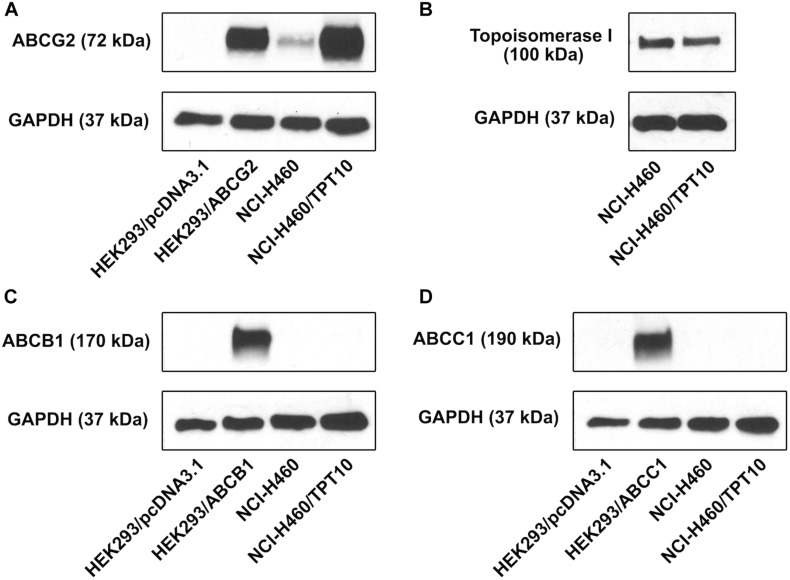
Protein expression profile of NCI-H460/TPT10 and parental NCI-H460 cells. Western blot on the expression levels of **(A)** ABCG2, **(B)** topoisomerase I, **(C)** ABCB1, and **(D)** ABCC1 in NCI-H460 and NCI-H460/TPT10 cells. HEK293/pcDNA3.1, HEK293/ABCG2, HEK293/ABCB1, and HEK293/ABCC1 cell lines served as negative or positive controls for ABCB1, ABCG2, and ABCC1 expression, respectively. The adopted loading control was GAPDH.

To investigate the drug resistant mechanism of NCI-H460/TPT10 cells, Western blotting analysis was performed to determine the protein expression levels of MDR-mediating ABC transporters, including ABCG2, ABCB1, and ABCC1, in both NCI-H460/TPT10 cells and parental NCI-H460 cells. Total cell lysate of HEK293/pcDNA3.1 was used as the negative control; meanwhile, positive controls of ABCG2, ABCB1, and ABCC1 expression were from cell lysates of HEK293/ABCG2, HEK293/ABCB1, and HEK293/ABCC1, respectively. As shown in Figure 1A, elevated ABCG2 expression was observed from the NCI-H460/TPT10 cell, exceeding that of the positive control HEK293/ABCG2, whereas NCI-H460 showed a low endogenous expression of ABCG2. While the expression of ABCB1 and ABCC1 were observed from the positive controls HEK293/ABCB1 and HEK293/ABCC1 cells, respectively, the expression of ABCB1 and ABCC1 were not observed in either NCI-H460 or NCI-H460/TPT10 (Figures 1C,D). Topoisomerase I (TOP1), as the target of topotecan (Pommier et al., 2010), was also investigated to ascertain whether drug target alternation is a mechanism of the drug-resistance in NCI-H460/TPT10 cell line. No significant difference between TOP1 expression levels in NCI-H460/TPT10 and NCI-H460 was shown (Figure 1B). Therefore, the overexpression of ABCG2 could be the main factor of the drug-resistance of NCI-H460/TPT10 after the long-term drug selection.

### Immunofluorescence Analysis

**FIGURE 2:**
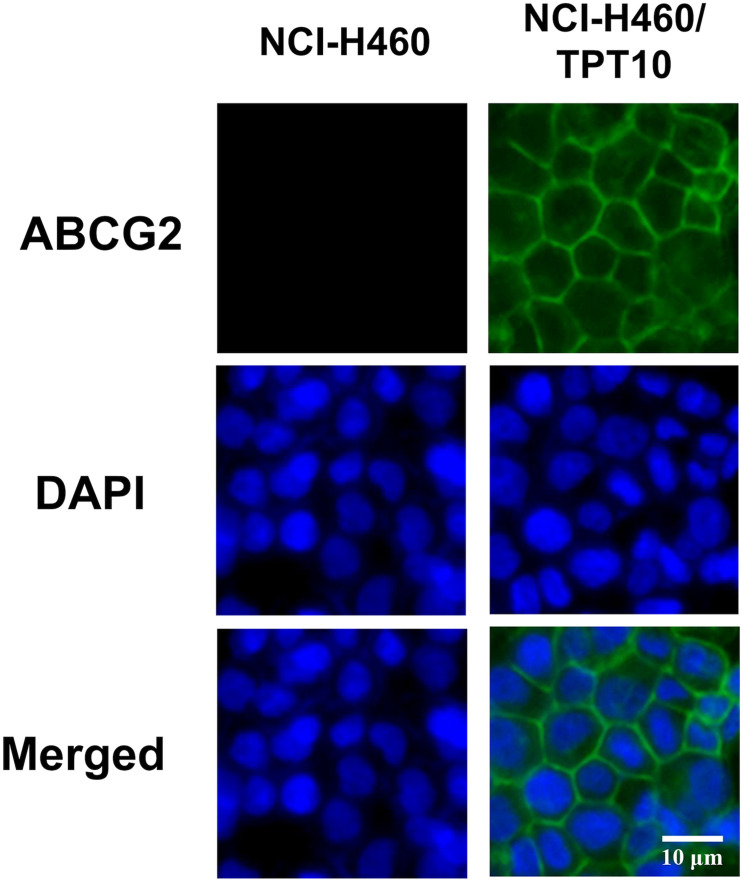
Immunofluorescence microscopic images of NCI-H460 and NCI-H460/TPT10 cells. ABCG2 and DAPI fluorescence micrographs were combined to create a merged image. ABCG2 expression was shown by green fluorescence; cell nuclei were stained blue by DAPI. This experiment has been done with triplicate wells in replicated independent tests.

To verify the overexpression of ABCG2 and its subcellular localization in NCI-H460/TPT10 cells, an immunofluorescence assay was performed (Figure 2). ABCG2 and DAPI fluorescence images were merged to show the localization of ABCG2. Overexpression of ABCG2 on plasma membranes were revealed in NCI-H460/TPT10 cells while the expression of ABCG2 of NCI-H460 cells was not detectable by immunofluorescence under the same staining condition and microscopic settings. This finding indicated that a potential drug resistant mechanism of NCI-H460/TPT10 may be the overexpression of ABCG2 localized on the cell membranes as efflux pump diminishing intracellular accumulation of anticancer drugs.

### Abolishment of Drug Resistance by ABCG2 Inhibitor and ABCG2 Gene Knockout in NCI-H460/TPT10 Cells

**FIGURE 3:**
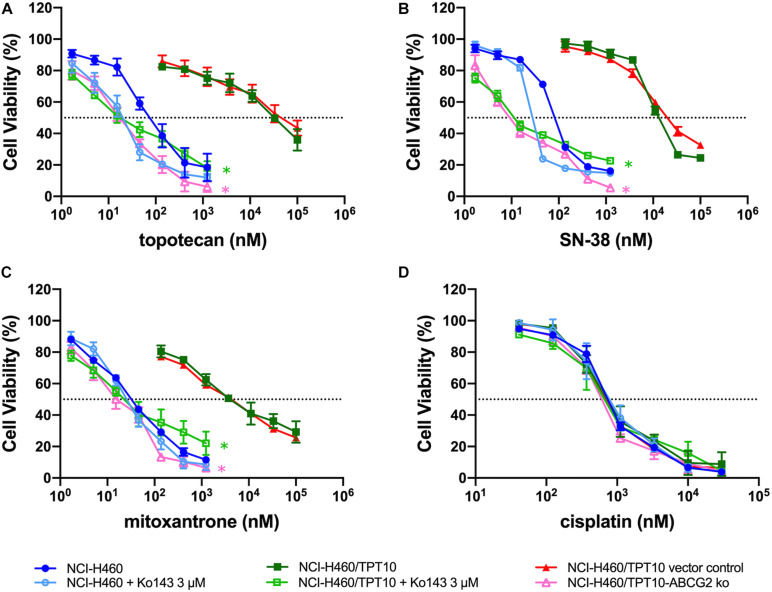
Abolishment of Drug Resistance by Ko143 and *ABCG2* gene Knockout in NCI-H460/TPT10 Cells. Cell viability was determined by MTT assay and displayed the changes in response to different concentrations of **(A)** topotecan, **(B)** SN-38, **(C)** mitoxantrone, and **(D)** cisplatin in drug resistant NCI-H460/TPT10 and the parental NCI-H460 cells, with or without 3 μM Ko143, and in NCI-H460/TPT10 ABCG2 knockout (ko) cells as well as the vector control subline. Data points with error bars represented the mean viability (%) ± SD of at least three independent experiments, each done in triplicate. Statistical analysis was performed to compare the IC_50_ values. * in green: *p* < 0.05 NCI-H460/TPT10 with Ko143 3 μM versus NCI-H460/TPT10 without Ko143. * in pink: *p* < 0.05 NCI-H460/TPT10-ABCG2 ko versus NCI-H460/TPT10 vector control.

To verify whether overexpression of ABCG2 is the major factor conferring the MDR characteristics in NCI-H460/TPT10 cells, Ko143, the known ABCG2 inhibitor, was inspected with the reversal effect. The concentration of Ko143 was selected based on its cytotoxicity to NCI-H460 and NCI-H460/TPT10 cells (Supplementary Figure 3). As shown in Figures 3A–C, Ko143 at 3 μM significantly re-sensitized NCI-H460/TPT10 cells to topotecan and the other two ABCG2 substrates, SN-38, and mitoxantrone, reflected by the notable shift of the dose-response curves to the left. The pre-treatment with Ko143 led to a significant drop in the IC_50_ values of ABCG2 substrates in NCI-H460/TPT10 cells to a level comparable to the IC_50_ values in NCI-H460, which suggesting that Ko143-mediated inhibition of the ABCG2 transporters resulted in a complete reversal of the drug-resistance. On the other hand, the IC_50_ values of ABCG2 substrates in the NCI-H460 cells did not undergo significant alternations by Ko143. Furthermore, the IC_50_ values of the non-ABCG2 substrate, cisplatin, had no change in both cell lines with the presence of Ko143 (Figure 3D).

Similar results were observed from NCI-H460/TPT10 cells with *ABCG2* gene knockout. Compared to the vector control, the NCI-H460/TPT10-ABCG2 knockout cells exhibited significantly reduced IC_50_ values of topotecan, SN-38 and mitoxantrone (Figures 3A–C), while the IC_50_ values of cisplatin were relatively consistent (Figure 3D), which confirmed the involvement of ABCG2 in MDR of NCI-H460/TPT10 cells.

### Accumulation of Topotecan in NCI-H460 and NCI-H460/TPT10 Cells

**FIGURE 4:**
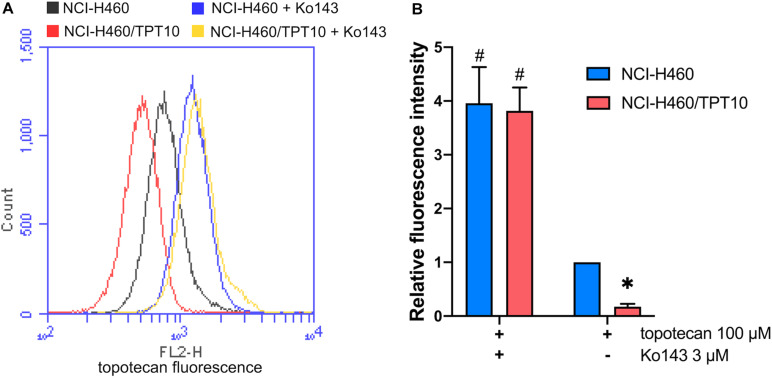
Topotecan accumulation in NCI-H460 and NCI-H460/TPT10 cells. **(A)** Flow cytometry detection of intracellular accumulation of topotecan in cells after 2-h exposure to 100 μM topotecan with or without 2-h pretreatment with 3 μM Ko143. **(B)** Intracellular topotecan accumulations in cells without Ko143 pretreatment are represented by the fold of fluorescence intensity. Fluorescence intensity of the accumulated topotecan in NCI-H460 cells without Ko143 was normalized to 1. Columns and error bars represented average values with SD from three independent measurements. * indicates *p* < 0.05 comparing resistant cell line with or without Ko143 to parental cell line with or without Ko143; # indicates *p* < 0.05 comparing a group with Ko143 to the corresponding cell line group without Ko143.

To further verify that the drug-resistance of NCI-H460/TPT10 cells was mainly due to an acquired capability to restrict intracellular topotecan accumulation by ABCG2 efflux transporter, it was considered necessary to evaluate and compare the intracellular topotecan accumulation levels between NCI-H460/TPT10 and parental NCI-H460 cells. NCI-H460/TPT10 cells exhibited reduced intracellular accumulation of topotecan compared to the parental NCI-H460 cells, whereas pre-treatment with 3 μM Ko143 elevated topotecan accumulation in both cell lines (Figure 4A). As illustrated in Figure 4B, functional inhibition of ABCG2 by Ko143 significantly increased the retention of topotecan in NCI-H460 and NCI-H460/TPT10 cells resulting in a similar accumulation level in both cell lines.

## Discussion

Chemotherapy is the predominant form of treatment for advanced NSCLC. Topotecan is one of the first-line drugs in treating NSCLC with a favorable side effect profile (Vennepureddy et al., 2015), but the development of MDR in tumors remains a serious obstacle for successful treatment. In MDR-exhibiting NSCLC cells that acquire resistance to one drug often develop resistance to a range of other structurally and functionally unrelated anticancer agents, resulting in cancer recurrence, and even relapse or death (Osmani et al., 2018). Thus, identifying the underlying mechanisms of drug resistance is critical for the development of new treatment strategies to overcome MDR and improve chemotherapeutic efficacy. In order to garner a broader understanding of the mechanisms of topotecan resistance in NSCLC, we established a topotecan-resistant NSCLC cell line by maintaining the parental NCI-H460 cells in stepwise increasing concentration of topotecan and termed this drug-resistant subline as NCI-H460/TPT10. The established cell line was first tested to confirm its resistance to topotecan, and the drug resistant profile was characterized. After a 5-month of selection with topotecan and 3 months culturing without topotecan, NCI-H460/TPT10 cells conferred a 394.7-fold resistance against topotecan compared to parental NCI-H460 cells, confirming the acquisition of topotecan resistance in the newly established cell line. Cross-resistance to SN-38 was shown in NCI-H460/TPT10 cells as expected since topotecan is a derivative of camptothecin, and SN-38 is the active metabolite of irinotecan, another camptothecin analog with a similar structure to topotecan. However, other structure unrelated drugs, such as mitoxantrone and doxorubicin, also had reduced cytotoxicity in NCI-H460/TPT10 cell line compared to its parental cell line, which suggested a potential involvement of ABC transporters in MDR of NCI-H460/TPT10 cells.

ABCB1, ABCC1, and ABCG2 are the three major ABC transporters present in MDR cancer cells, and each of them has a broad substrate range overlapping with the other two. For instance, mitoxantrone and doxorubicin are known substrates of ABCB1, ABCC1 as well as ABCG2, and topotecan has been reported to be an overlapping substrate of ABCB1 and ABCG2 (Stacy et al., 2013). Therefore, the expression level of ABCB1, ABCC1, and ABCG2 were examined by Western blotting in the newly established NCI-H460/TPT10 cells. It has been suggested that topotecan resistance in some types of cancers is related to the overexpression and active drug efflux functions of ABCB1 and ABCG2. Cross-resistant to topotecan has been observed in ABCB1-overexpressing human ovarian cancer cell lines (Januchowski et al., 2016). Topotecan selected drug resistance was found related to expression and efflux activity of ABCG2 expression in human breast cancer cells (Yang et al., 2000) or overexpression of both ABCG2 and ABCB1 transporters in human ovarian cancer cells (Januchowski et al., 2016). In this study, overexpression of ABCG2, but no detectable expression of ABCB1 or ABCC1, was observed in NCI-HC460/TPT10 cells, suggesting that ABCG2 is a promising candidate responsible for the resistance of topotecan and other ABCG2 substrates in NCI-H460/TPT10 cells. This also explained the observations that NCI-H460/TPT10 cells displayed no resistance to non-ABCG2 substrates such as paclitaxel, colchicine, vincristine, and cisplatin. The relative low resistant fold showed when comparing the doxorubicin IC_50_ values between parental and the new resistant cell lines might be due to the fact that doxorubicin is a potent substrate of ABCB1 but a relatively weak substrate of ABCG2 (Heyes et al., 2018).

Topotecan exerts anticancer effects by targeting DNA topoisomerase I and blockade of DNA replication (Li et al., 2017). As NCI-H460/TPT10 cell line was established by topotecan selection, there might be an alternation of topoisomerase I expression as a consequence. Down-regulation of topoisomerase I has been observed in topotecan-resistant human SCLC cells derived from the OC-NYH cell line (Sorensen et al., 1995) and in topotecan-resistant human breast cancer cells established from MCF-7 cell line (Yang et al., 2000). However, in these studies, it has been suggested that the decreased level of topoisomerase I had only partial or no contributions to the MDR phenotype in the topotecan-resistant cells. Another study reported that in an established topotecan-resistant cell line on the basis of human IGROV-1 ovarian cancer cell line, no expressional and functional changes of topoisomerase I protein were observed, indicating that the drug resistance and reduced intracellular topotecan accumulation was not related to topoisomerase I (Ma et al., 1998). Similarly, the Western blotting results in this study showed no remarkable difference in the protein expression of topoisomerase I between the drug resistant NCI-H460/TPT10 and the parental NCI-H460 cells. The cell division is also related to topotecan resistance as topotecan targets the topoisomerase I-DNA complex. It could be assumed that fast-dividing cells may be more drug sensitive to topoisomerase inhibitors than slowly dividing cells (Tesauro et al., 2019). As observed in this study, the PDT of NCI-H460/TPT10 was extended compared to the parental cell line but the change was insignificant, which indirectly indicated that the drug resistant mechanism may not reply on change in topoisomerase activity. Therefore, the main mechanism responsible for topotecan resistance in NCI-H460/TPT10 cells was more likely to be the active removal of topotecan from the cells via its overexpression of ABCG2.

ABCG2 usually acts as a homodimer or an oligomer to distribute on plasma membrane expelling substrate drugs as it’s a half transporter which has one transmembrane domain (TMD) and one nucleotide-binding domain (NBD) (Taylor et al., 2017). Our immunofluorescence imaging results confirmed that the high expression of ABCG2 was majorly distributed on the plasma membrane of the drug resistant cells NCI-H460/TPT10, leading to a hypothesis that the overexpression of ABCG2 transporter on cell membrane functions to pump out the intracellular anticancer drugs thereby resulting in drug resistance in NCI-H460/TPT10 cells. This hypothesis was further verified by accessing the reversal effect of a potent ABCG2 inhibitor, Ko143, and the abolishment of drug resistance by *ABCG2* gene knockout in NCI-H460/TPT10 cells. Pre-treatment with Ko143 at a non-toxic concentration (3 μM) significantly re-sensitized NCI-H460/TPT10 cells to topotecan and other ABCG2 substrate drugs, with IC_50_ values comparable to those in the drug-sensitive NCI-H460 cells. Additionally, the remarkable diminished topotecan accumulation in NCI-H460/TPT10 cells could be restored by inhibiting ABCG2 using Ko143, which indicated that the drug resistance of NCI-H460/TPT10 could be completely reversed by inhibiting the drug efflux function of ABCG2 function. Besides of functional inhibition of ABCG2, loss of ABCG2 protein expression by gene knockout also abolished the MDR feature of NCI-H460/TPT10 cells. These findings validate that the elevated protein expression of ABCG2 is the leading cause of drug resistance in NCI-H460/TPT10 cells. As there is endogenous low ABCG2 expression in parental NCI-H460 cells, a slight decrease in IC_50_ of ABCG2 substrates and a notable increase in intracellular topotecan accumulations were also observed in NCI-H460 cells pre-treated with Ko143.

In summary, the new-established NCI-H460/TPT10 cell line is useful for studying ABCG2-mediated MDR and other topotecan-related resistance mechanisms in NSCLC. Although the data in our study suggested that the elevated expression of ABCG2 on the membrane of NCI-H460/TPT10 cells is the major factor accounting for its MDR phenotype, much is still unknown about ABCG2 transport substrate interaction on the molecular level. The established models in this study can be useful for in-depth investigation of these interactions, which will be crucial for future drug design. The NCI-H460/TPT10 cell line has been proved to be tumorigenic *in vivo* in the preliminary experiment of the other project of our team (Supplementary Figure 4). Future research will focus on further validation of this model in *in vivo* systems, elucidation of the detailed MDR mechanisms, and screening for potential effective agents to reverse drug resistance.

## Data Availability Statement

The original contributions presented in the study are included in the article/Supplementary Material, further inquiries can be directed to the corresponding author/s.

## Author Contributions

Z-NL, Q-XT, WZ, and Z-SC designed the experiments. Z-NL, Q-XT, WZ, Y-FF, J-QW, C-YC, and KL performed the experiments. WZ, D-HY, and JW provided the technical and material support. Z-SC and D-HY reviewed and revised the manuscript. All authors discussed the results and implications and commented on the manuscript at all stages.

## Conflict of Interest

The authors declare that the research was conducted in the absence of any commercial or financial relationships that could be construed as a potential conflict of interest.
