# Supplementary material for: Establishment and Characterization of a Topotecan Resistant Non-small Cell Lung Cancer NCI-H460/TPT10 Cell Line
Source: Front Cell Dev Biol. 2020 Dec 23;8:607275. doi: 10.3389/fcell.2020.607275 (PMC7786180; doi:10.3389/fcell.2020.607275)
Supplement: Supplementary file 1 [file Data_Sheet_1.pdf]

Supplementary Material

1 Supplementary Figures

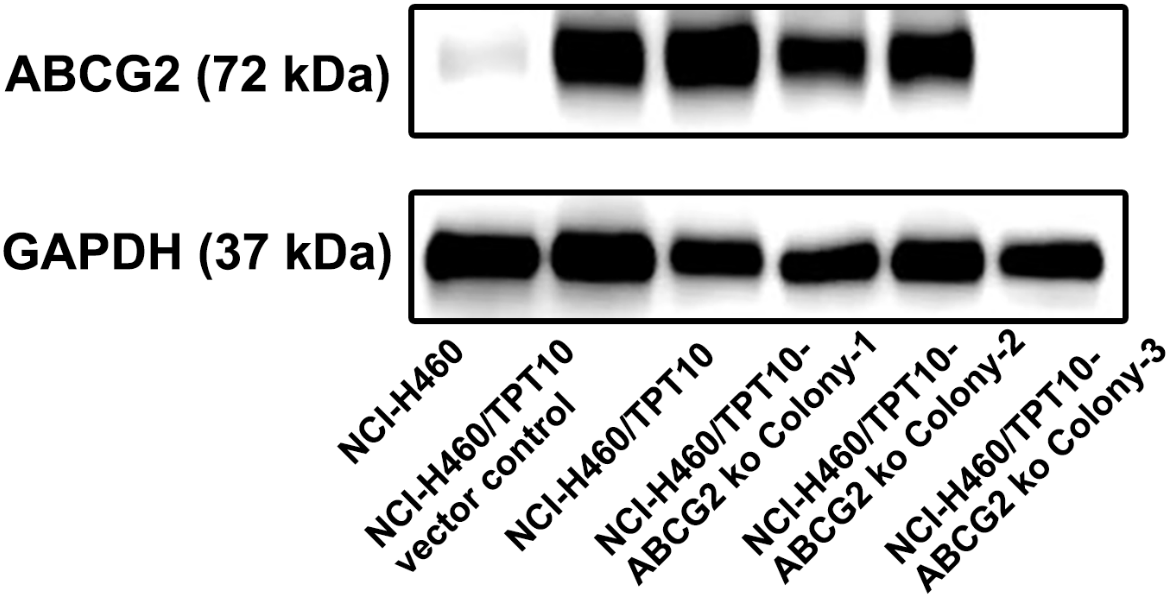

Figure S1. Verification of NCI-H460/TPT10-ABCG2 knockout (ko) subline. Western blot on the expression levels of ABCG2 in NCI-H460, NCI-H460/TPT10, NCI-H460/TPT10 vector control and three different colonies obtained from NCI-H460/TPT10 cells transfected with the CRISPR plasmid targeting *ABCG2* gene. GAPDH was used as a loading control. Colony-3 was selected as positive *ABCG2* gene knockout subline of NCI-H460/TPT10 and was used for MTT assay to determine drug sensitivity as described in section "Construction of NCI-H460/TPT10 ABCG2 Knockout Cell Line".

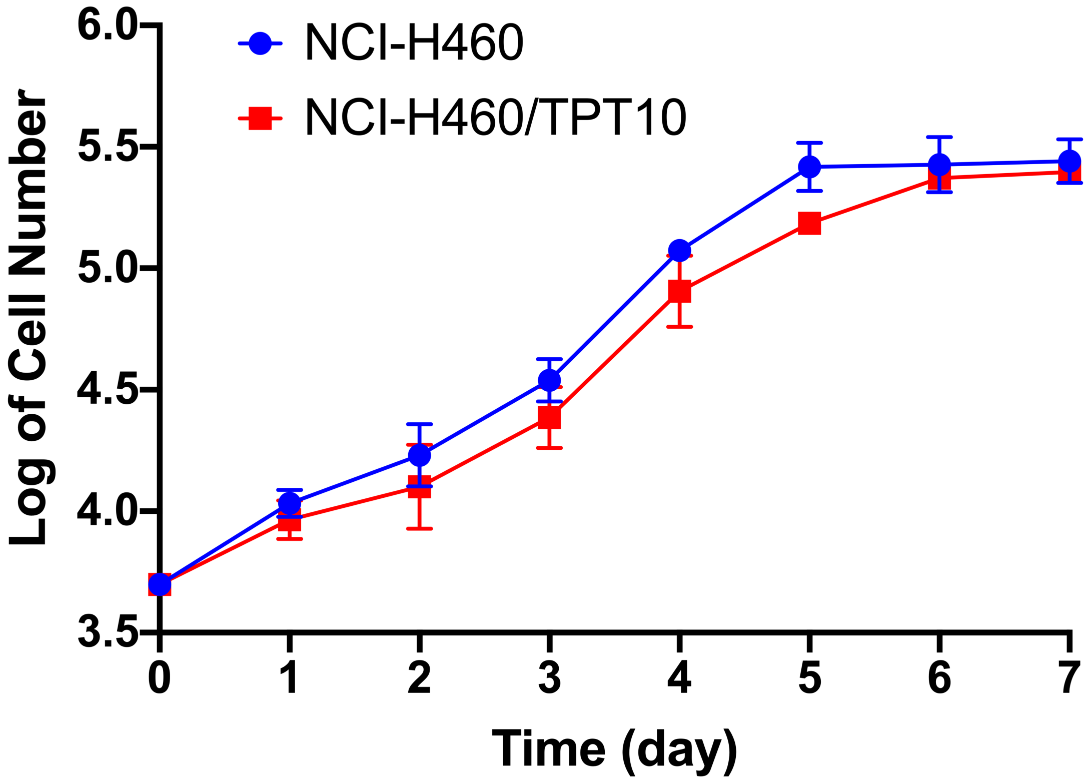

Figure S2. The cell growth curves of NCI-H460 an NCI-H460/TPT10 cell lines. Triplicate cell counts using trypan blue exclusion method for each cell line were made every 24 h for 7 days. Data points represented the logarithm of mean cell number with a base of 10, and error bars represent the SD. Data were collected from three independent experiments.

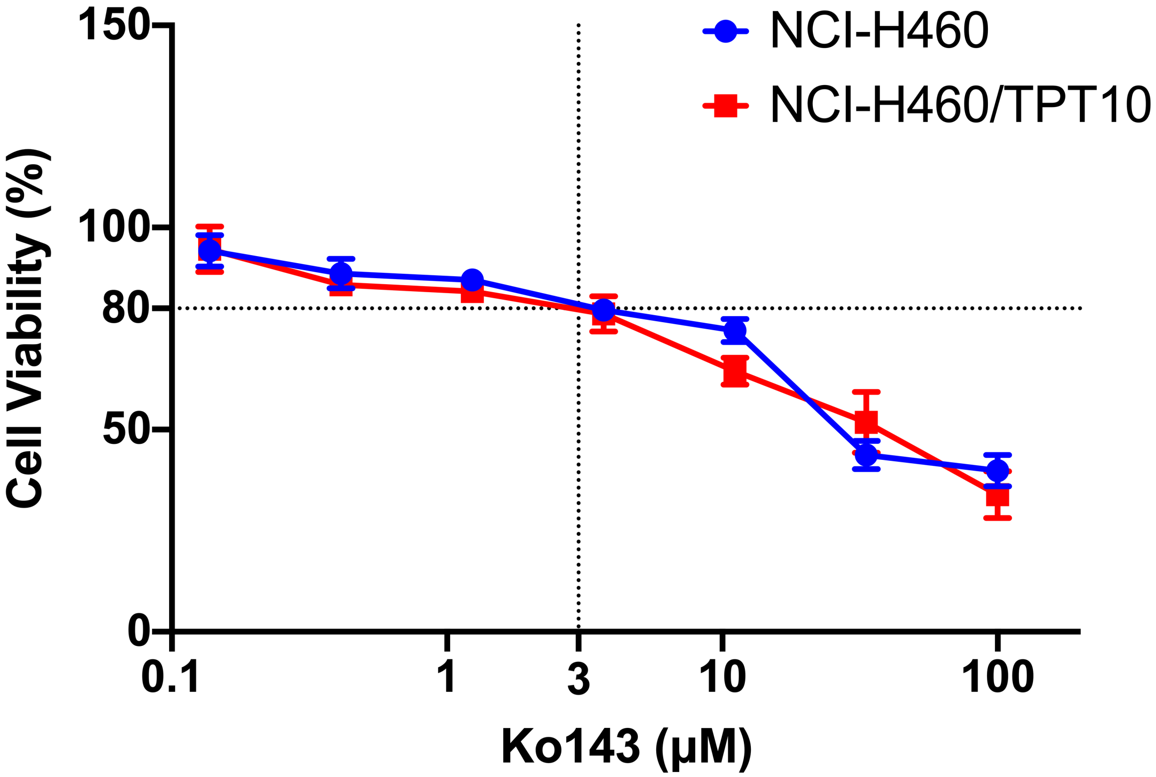

Figure S3. Cytotoxicity of Ko143 to NCI-H460 and NCI-H460/TPT10 cells. Cell viability was determined by MTT assay and displayed the changes in response to different concentrations of Ko143. The concentration of 3 μM, at which at least 80% cells survived, was selected for the reversal study where Ko143 was used as an ABCG2 inhibitor.

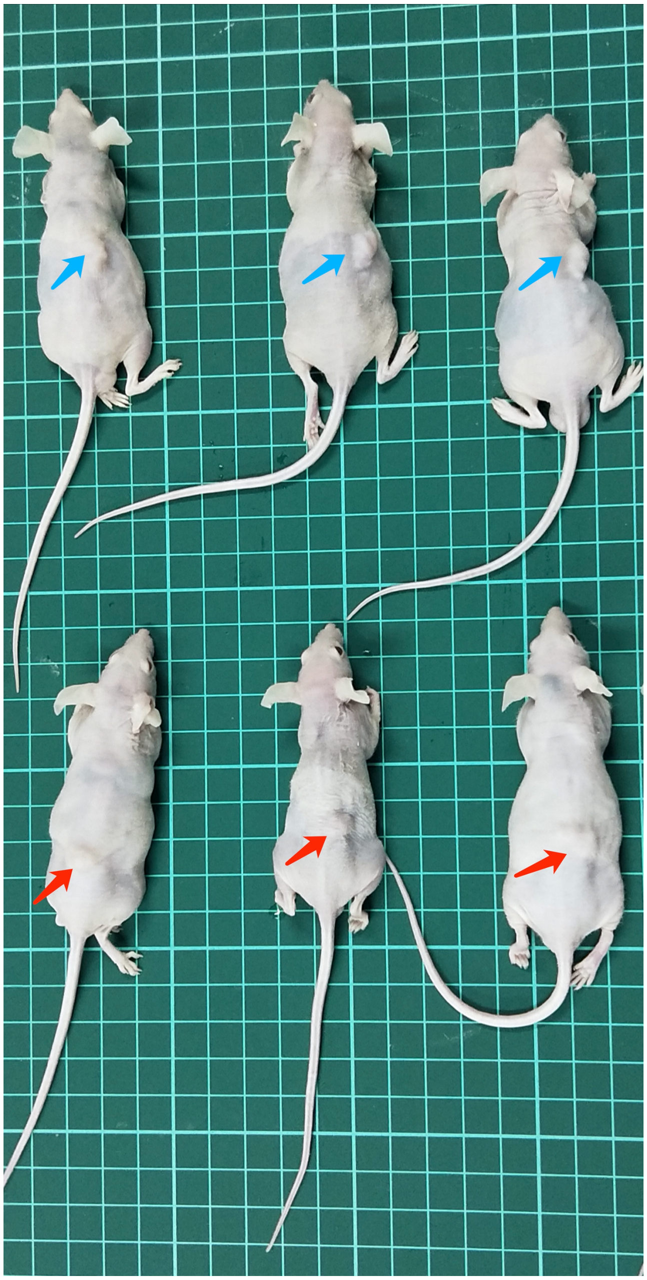

Figure S4. Verification of tumorigenicity of NCI-H460 and NCI-H460/TPT10 cell lines. The image of tumor xenografted athymic NCR nude mice on day 10 after subcutaneously implanted with NCI-H460 (pointed with blue arrows) or NCI-H460/TPT10 (pointed with red arrows) cell line into the back.

2 Supplementary Table

Table S1. The STR profile analysis of NCI-H460 and NCI-H460/TPT10 cell lines by ATCC cell line authentication service.

Loci	NCI-H460 from the ATCC STR Profile Database	NCI-H460	NCI-H460/TPT10
D5S818	9,10	9,10	9,10
D13S317	13	13	13
D7S820	9,12	9,12	9
D16S539	9	9	9
vWA	17	17	17
TH01	9.3	9.3	9.3
Amelogenin	X,Y	X,Y	X,Y
TPOX	8	8	8
CSF1PO	11,12	11,12	11,12
% Match[a]	-	100%	92%

a % Match compared to the STR profile of NCI-H460 from the ATCC STR Profile Database. Cell lines with more than 80% match are considered to be related, i.e., derived from a common ancestry.
